# Molecular Imaging of Brain Tumors and Drug Delivery Using CEST MRI: Promises and Challenges

**DOI:** 10.3390/pharmaceutics14020451

**Published:** 2022-02-20

**Authors:** Jianpan Huang, Zilin Chen, Se-Weon Park, Joseph H. C. Lai, Kannie W. Y. Chan

**Affiliations:** 1 Department of Biomedical Engineering, City University of Hong Kong, Hong Kong, China; jp.huang@cityu.edu.hk (J.H.); zilinchen9-c@my.cityu.edu.hk (Z.C.); swpark3-c@my.cityu.edu.hk (S.-W.P.); josephlai5-c@my.cityu.edu.hk (J.H.C.L.); 2 Hong Kong Centre for Cerebro-Cardiovascular Health Engineering (COCHE), Hong Kong, China; 3 Russell H. Morgan Department of Radiology and Radiological Science, The Johns Hopkins University School of Medicine, Baltimore, MD 21205, USA; 4 Shenzhen Research Institute, City University of Hong Kong, Shenzhen 518057, China; 5 Tung Biomedical Science Centre, City University of Hong Kong, Hong Kong, China

**Keywords:** CEST, MRI, molecular imaging, brain tumor, contrast agents, chemotherapeutics, drug delivery

## Abstract

Chemical exchange saturation transfer (CEST) magnetic resonance imaging (MRI) detects molecules in their natural forms in a sensitive and non-invasive manner. This makes it a robust approach to assess brain tumors and related molecular alterations using endogenous molecules, such as proteins/peptides, and drugs approved for clinical use. In this review, we will discuss the promises of CEST MRI in the identification of tumors, tumor grading, detecting molecular alterations related to isocitrate dehydrogenase (IDH) and O-6-methylguanine-DNA methyltransferase (MGMT), assessment of treatment effects, and using multiple contrasts of CEST to develop theranostic approaches for cancer treatments. Promising applications include (i) using the CEST contrast of amide protons of proteins/peptides to detect brain tumors, such as glioblastoma multiforme (GBM) and low-grade gliomas; (ii) using multiple CEST contrasts for tumor stratification, and (iii) evaluation of the efficacy of drug delivery without the need of metallic or radioactive labels. These promising applications have raised enthusiasm, however, the use of CEST MRI is not trivial. CEST contrast depends on the pulse sequences, saturation parameters, methods used to analyze the CEST spectrum (i.e., Z-spectrum), and, importantly, how to interpret changes in CEST contrast and related molecular alterations in the brain. Emerging pulse sequence designs and data analysis approaches, including those assisted with deep learning, have enhanced the capability of CEST MRI in detecting molecules in brain tumors. CEST has become a specific marker for tumor grading and has the potential for prognosis and theranostics in brain tumors. With increasing understanding of the technical aspects and associated molecular alterations detected by CEST MRI, this young field is expected to have wide clinical applications in the near future.

## 1. Introduction

Brain tumors are hard to diagnose early and treat. Advances in genomics have enabled the study of molecular alterations in tumors which is critical for the diagnosis and prognosis of brain tumors, as well as to guide treatments. Glioblastoma multiforme (GBM) is the most lethal form of brain tumors. Many related molecular alterations have been found, such as epidermal growth factor receptor (EGFR), isocitrate dehydrogenase (IDH), and O-6-methylguanine-DNA methyltransferase (MGMT) [1]. These alterations do not only vary among different types of brain tumors, the level of alterations is also heterogenous within tumors. Historically, brain tumor diagnosis is based on histologic features. The inclusion of new multiple assessment parameters could address challenges in brain tumor diagnosis, treatment planning, and evaluation. Some of these molecular alterations have been incorporated into the diagnostic criteria of brain tumors [2]. For example, IDH mutation is one of the assessment criteria in diffusive gliomas and astrocytoma. MGMT has been found to associate with improved response to treatment with temozolomide (TMZ) and longer overall survival [2]. Imaging is an indispensable way to address the heterogeneity within tumors. Chemical exchange saturation transfer (CEST) is an emerging molecular imaging approach which enables the assessments of molecular alterations in brain tumors [3,4,5,6,7,8,9,10,11,12,13,14,15,16,17,18,19,20,21,22,23,24,25,26,27,28,29,30,31,32,33,34,35,36,37,38,39,40,41,42,43,44,45].

**Figure 1:**
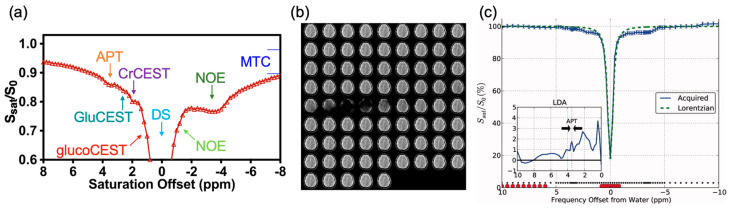
(**a**) Schematic illustration of the Z-spectrum with multiple CEST contrast. APT: amide proton transfer; GluCEST: glutamate CEST; CrCEST: creatine CEST; glucoCEST: glucose CEST; DS: direct water saturation; NOE: nuclear Overhauser effect; MTC: magnetization transfer contrast. (**b**) Saturated images as a function of saturation frequency for a human brain slice. (**c**) In vivo Z-spectra and Lorentzian difference analysis (LDA) for a region in the white matter of the human brain. Figure panels (**b**,**c**) are reproduced with permission from Jones et al. Magn Reson Med 2012;67(6):1579–1589. Copyright John Wiley and Sons, 2012.

CEST MRI was named by Ward and Balaban in 2000 [46]. It detects exchangeable protons of molecules, such as amide protons of proteins/peptides (amide proton transfer, APT at 3.5 ppm) [3,47,48], aliphatic protons of lipids (nuclear Overhauser effect, NOE at −3.5 ppm) [43,44], hydroxyl protons (e.g., glucose at 1–2 ppm), and guanidyl protons (e.g., creatine, at 2 ppm) [49,50,51]. These exchangeable protons can be detected via the acquisition of a CEST spectrum, i.e., Z-spectrum (Figure 1a,c). CEST is capable of detecting multiple molecules in the brain (Figure 1b,c) such as macromolecules (magnetization transfer contrast, MTC), semi-solids (APT and NOE), and metabolites (e.g., glucose and creatine) simultaneously. Further CEST principles can be found in reviews [49,50,51,52]. Magnetic resonance spectroscopy (MRS) is a conventional molecular imaging approach. Its clinical applications have been limited by relatively low sensitivity and low spatial resolution. CEST MRI detects molecules via proton exchange with bulk water (110 M) which is abundant in vivo [47,50]. It has an imaging readout that enables relatively high-resolution detection of the spatial distribution of molecules. Thus, CEST could address the clinical needs in molecular imaging. Notably, MRS detects the distinct chemical environments of molecules while CEST detects exchangeable protons and their exchange environment [53,54]. Nevertheless, detecting molecular alterations and their location is invaluable in assessing brain tumors, especially when more than one molecule is altered in brain tumors in various regions [55,56,57,58].

CEST MRI has been applied to grade tumors [5,8,9,19,22,23,27,30,31,32,33,39,59,60,61,62,63,64,65], assess treatment effects [13,14,21,32,40,66,67,68,69,70], assess progression survival [13,16,17], and evaluate IDH mutation [5,9,17,20,22,24,29,71,72]. Furthermore, the molecular and regional changes in tumors revealed by CEST could inform the development of theranostic approaches for brain tumors, especially for monitoring the changes of multiple components of drug delivery systems longitudinally. This review will focus on the promises of CEST imaging of brain tumors, non-metallic contrast agents, clinical agents and biomaterials for drug delivery, and the challenges of CEST clinical applications.

## 2. CEST Imaging of Brain Tumors

### 2.1. Endogenous Contrast

CEST is capable of detecting the presence of millimolar concentrations of molecules in vivo. The two unique CEST contrasts at 3.5 ppm (APT) and −3.5 ppm (NOE) are widely used to assess brain tumors [5,8,9,13,14,16,17,19,20,21,22,23,24,27,29,30,31,32,33,39,40,59,60,61,62,63,64,65,66,67,68,69,70,71,72]. Tumors have an acidic microenvironment and CEST is sensitive to pH; hence, CEST could further enhance the detectability of these molecular alterations in tumors [3,58,73]. The principles of CEST and APT imaging have been explained in previous reviews [7,49,50,51,52,74,75,76,77,78,79]. APT and NOE changes could indicate changes in protein/lipid concentration, pH, and cellularity. CEST imaging studies of brain tumors in both animals and humans are summarized in Table 1. With more clinical studies available for GBM and other gliomas [1,2,80], both APT and NOE could provide valuable molecular information for brain tumor assessments, especially towards precise diagnosis and prognosis.

#### 2.1.1. APT-Weighted (APTw) Contrast

CEST contrast at 3.5 ppm could be characterized using the conventional magnetization transfer ratio asymmetry (MTR_asym_) analysis of the Z-spectrum, subtracting signals at 3.5 ppm from −3.5 ppm frequency offsets. There was not much of a magnetization transfer (MT) effect in solutions in vitro [47], while an asymmetric MT effect was observed in cross-linked bovine serum albumin (BSA) and semi-solid protein-rich phantoms [81,82,83,84,85]. As demonstrated in early animal studies in 2003, amide proton exchange characterized by MTR_asym_ (APTw) resulted in hyperintensity in the 9L brain tumor rat model [3,48] which could be ascribed to the elevated cellular proteins and peptides in tumors. Moreover, APTw is robust in the identification of radiation necrosis from tumor recurrence [70,86]. The reduced APTw signal after radiation therapy indicated a molecular tumor response that was detected earlier than other commonly used MRI approaches [70]. APTw CEST was then translated to examine the human brain in 2006 [4].

In human patients, hyperintensities of APTw were found in tumors when compared with the contralateral regions [11,23,25,26,33,36,39,42,43,44,60,70,87,88,89,90,91,92] (Table 1). These increased APTw signals were positively correlated to high cellularity [39,70] and were validated by histology. The APTw signal in the core of high-grade tumors was much higher than that of low-grade tumors (2.7 ± 0.3% vs. 1.2 ± 0.2%, *n* = 6 and 3, respectively), demonstrating a great potential of APTw imaging in tumor grading on a 3 T clinical scanner [60]. Another study of diffuse glioma patients (*n* = 36) [39] also reported a significant correlation between APTw signal and tumor grade. Specifically, APTw signal was 2.1 ± 0.4% (*n* = 8), 3.2 ± 0.9% (*n* = 10), and 4.1 ± 1.0% (*n* = 18) for grade II, III, and IV gliomas, respectively. Moreover, the authors suggested a cutoff APTw value of 2.54% to differentiate high-grade tumors from low-grade tumors with a sensitivity of 93% and specificity of 100%. Similarly, APTw signal increased with tumor grade in diffuse glioma patients (*n* = 46) and an improvement was found by using APTw and apparent diffusion coefficient (ADC) to grade tumors compared to using ADC only (AUC: 0.910 vs. 0.888) [31]. The ability of APTw imaging to identify high- and low-grade gliomas were found to be efficient even using a single representative slice for analysis (*n* = 26) [65]. All these studies demonstrated the great potential of using the APTw signal to grade brain tumors in clinical settings.

#### 2.1.2. NOE Contrast

Brain tumors have a complicated microenvironment and multiple molecules could contribute to the resulting APTw contrast. When we consider CEST at 3.5 ppm and −3.5 ppm independently, the observations are slightly different. CEST at 3.5 ppm alone corrected using the apparent exchange-dependent relaxation (AREX) did not show a significant change in a rat tumor model at 9.4T which was validated by biochemical means [93]. Moreover, the corrected NOE signal showed a significant decrease in the tumor than that in normal tissue [93]. Therefore, it is also valuable to investigate the NOE signal change in tumors. The NOE contrast has a broad range of frequency offsets, from −1.5 to −5 ppm [33,44,81,94,95,96]. Most of the studies focused on the offset at around −3.5 ppm as it is more detectable, especially at 3 T [81,97]. NOE at −3.2 to −3.7 ppm, extracted using Lorentzian fitting, showed decreased signals in brain tumors in both animal and human studies [10,33,34,44,90,98,99,100]. NOE analyzed by AREX showed hypointensity in brain tumors as compared to contralateral regions which correlated to the macromolecular content in tumors [96]. Heo et al. found that the NOE signal showed a negative correlation with tumor grades [33]. This indicates the feasibility of grading brain tumors using NOE imaging. Recently, Zu Z. et al. observed a decrease in NOE signal at −1.6 ppm, which could indicate decreased phospholipids on tumor cell membranes, by restricted phospholipid transfer (RPT) [101]. In general, NOE signals consistently decrease among different analyses in both human and animal studies. Among these studies, there is one study that showed unchanged NOE in a non-enhancing glioma, while APTw signal consistently increased in both enhancing and non-enhancing gliomas [11]. Nevertheless, NOE could be an additional CEST contrast to indicate molecular changes in brain tumors.

### 2.2. Glioblastoma and Gliomas (Grade II, III)

GBM, also known as a grade IV astrocytoma, defined by World Health Organization (WHO), is the most common and aggressive brain tumor. Generally, hyperintensities in APT/APTw images were reported in GBM when compared with contralateral regions [10,11,16,22,25,33,34,36,39,42,60,98]. In human GBM, the increased signals were found to correlate positively to cell proliferation (Ki-67) and cell density, validated by histology [27,39]. The hyperintensity of APTw in tumors was related to high tumor grade and active cell proliferation [39,70,88]. Naturally, hypercellularity leads to an increase in cytosolic protein/peptides in tumor tissues as compared to normal tissues [102,103]. Thus, there is a positive correlation between the APTw signal and cytosolic protein/peptides concentration [90]. However, this might not be held in some tumor regions [90,102,104].

In addition to protein concentration, pH is another factor that attenuates the APT signal via altering the exchange rate. According to previous studies using ^31^P NMR spectroscopy, the intracellular pH of GBM is neutral or slightly alkaline with a minor increase of less than 0.1 pH unit [105,106]. On the other hand, a small change of pH may alter the APT signal since the amide proton exchange process is base-catalyzed [90,104,107,108]. The extravascular and extracellular space (EES) of tumors is acidic. A study reported that the contribution of protein concentration and pH change is 66% and 34% in tumors, respectively [108].

The mobility of proteins and peptides is another factor that could contribute to the APTw signal [75]. The liquefactive necrosis and chronic hemorrhage in high-grade brain tumors resulted in an increased APTw signal [45], which was validated by other MRI readouts, such as FLAIR and T1w. The acute hemorrhage showed a higher APTw signal compared to a subacute hemorrhage, while a high APTw signal related to hemorrhage was observed in both tumorous and non-tumorous regions [109]. This hyperintensity of the APTw signal caused by hemorrhage and vascularity was then validated in a rat model [110]. These studies indicated that a high concentration of mobile proteins and peptides in liquid-like necrosis, hemorrhage, and vascularity could also lead to a high APTw signal. Additionally, the viable tumor core had a higher APTw signal than tumor necrosis and normal tissue [45,70]. Therefore, the interpretation of an increase in APTw signal should be cautious and consider the heterogeneity of brain tumors.

Brain tumors are heterogeneous and have different grades. Hence, it is not surprising to observe characteristic regional changes. For example, the APTw changes in dynamic contrast enhancing (DCE) enhancing and non-enhancing tumors could be different [45,111], since there is a higher water content in tumors as compared to normal brain tissues [90]. The enhancing region of high-grade tumors is usually associated with high cell density. Moreover, a decreased ADC value [16,34], intratumoral necrosis [39] and relative cerebral blood volume (CBV) [16] were also observed in GMB. These changes further validated that CEST contrast at 3.5 ppm sensitively detects molecular changes in GBM related to its aggressiveness. Meanwhile, CEST at 3.5 ppm showed an increased signal in brain tumors with other analyses such as direct saturation-corrected (DISC-CEST) [26,92], three-offset analysis [26], and quasi–steady-state (QUASS) [10]. More neuropathologies could be revealed when combining T1 and diffusion findings in APT interpretation.

GBM has the highest APTw signal among astrocytomas at different grades, including anaplastic astrocytoma at WHO grade III and astrocytoma at grade II [22,39,42,60], indicating CEST contrast could assist in astrocytoma grading. IDH mutation is mainly found in grade II/III gliomas and secondary GBM [112,113]. IDH gene-encoded enzymes participate in several cellular functions, such as amino acid metabolism, lipid metabolism, and genome-wide DNA methylation. IDH mutation and the MGMT promoter methylation have been included as critical prognostic molecular markers for glioma [114]. CEST at 3.5 ppm demonstrated hyperintensity in IDH-wild type when compared with IDH-mutant glioma patients, along with a high level of relative CBV [16,22,29], while showed no significant differences regarding the MGMT promoter methylation status [22]. NOE imaging related to this mutation is quite diverse [5,22]. Overall, CEST at 3.5 ppm may serve as the IDH mutation marker and help in glioma status prediction.

**Table 1:** CEST MRI of brain tumors using endogenous contrast.

Species	Tumor Type (Grade)	B_0_ (T)	Analysis Method	CEST Contrast	Molecular/Cellular Changes	Ref.
Rat	Glioma, C6	3	DISC-CEST	APT	Cellular and nuclear atypia	Wu Y. et al., 2019 [92]
Rat	Gliosarcoma, 9L	4.7	MTR_asym_	APTw	Cellular proteins and peptides	Zhou Z. et al., 2003 [3]
Rat	Gliosarcoma, 9L	4.7	MTR_asym_	APTw	pH	Zhou Z. et al., 2003 [48]
Rat	Gliosarcoma, 9L SF188/V + glioma	4.7	MTR_asym_	APTw	Treatment effects (radiation therapy), radiation necrosis, mobile cytosolic proteins, and peptides	Zhou J. et al., 2011 [70]
Rat	Gliosarcoma, 9L	4.7	MTR_asym_	APTw NOE (−2.5 to −5 ppm)	Mobile proteins, peptides, lipids, and metabolites	Zhou J. et al., 2013 [43]
Rat	U87	4.7	MTR_asym_	APTw	Treatment effects (radiation therapy), radiation necrosis, cellularity, nuclear atypia, and vacuolation	Hong X. et al., 2014 [69]
Rat	GBM	4.7	EMR	APT, NOE	Mobile proteins and peptides	Heo HY. et al., 2016 [87]
Rat	GBM	4.7	MTR_REX_, AREX, CESTR, CESTR^nr^	APT, 2 ppm	APT: mobile proteins and peptides, 2 ppm: protein and peptide side-chain amide protons and various amine-related protons	Heo HY. et al., 2017 [89]
Rat	U87	4.7	MTR_asym_	APTw	Amide proton mobile amide proton content or the increased amide proton exchange rate	Lee DH. et al. 2017 [90]
			EMR	APT, NOE		
Rat	Glioma	4.7	DISC-CEST	APT NOE	APT: intracellular mobile proteins/peptides concentration NOE: aliphatic and olefinic protons	Zhou IY. et al., 2017 [26]
Rat	Gliosarcoma, 9L	4.7	MTR_asym_	APTw	NA	Heo H. et al., 2019 [91]
			EMR	APT, NOE		
Rat	Gliosarcoma, 9L	9.4	AREX	APT, NOE	Protein contents	Xu J. et al., 2014 [93]
Rat	Gliosarcoma, 9L	9.4	Lorentzian	APT (3.6 ppm) NOE (−3.2 ppm)	Amide proton	Cai K. et al., 2015 [115]
				2 ppm	Tumor progression and creatine	
Rat	Gliosarcoma, 9L; glioma, F98	9.4	Lorentzian	2 ppm	Creatine and tumor aggressiveness	Cai K. et al., 2017 [116]
Rat	Gliosarcoma, 9L	9.4	MTR_asym_, AREX	3 ppm	Amine and protein	Zhang XY. et al., 2017 [117]
Rat	ENU1564 (brain metastasis model)	9.4	APTR*	APT	Protein concentration and pH	Ray KJ. et al., 2019 [107]
Rat	Gliosarcoma, 9L	9.4	Lorentzian	3 ppm	Glutamate	Debnath A. et al., 2020 [118]
Rat	Gliosarcoma, 9L	9.4	RPT	NOE (−1.6 ppm)	Phospholipids on cell membranes	Zu Z. et al., 2020 [101]
Mouse	GBM, patient cells	7	MTR_asym_	APTw	Proliferation, cellular acidification, and treatment effect (TMZ)	Sagiyama K. et al., 2014 [40]
Mouse	Glioma, GL261	7	MTR_asym_	3 ppm	Amine, pH, cellularity, and necrosis	Harris RJ. et al., 2015 [38]
Mouse	U87MG	9.4	AACID	AACID (amide at 3.5 ppm, amine at 2.75 ppm)	Intracellular pH and treatment effect	Albatany M. et al., 2019 [66]
Human (*n* = 10)	GBM (IV), oligodendroglioma (III), LGO (II), LGA (II), Meningioma	3	MTR_asym_	APTw	Cellular protein/peptide and intracellular pH	Jones CK. et al., 2006 [4]
Human (*n* = 9)	GMB (IV), AO (III), AA (III), LGO (II), LGA (II)	3	MTR_asym_	APTw	Glioma grading, cytosolic protein and peptide, and intracellular pH	Zhou J. et al., 2008 [60]
Human (*n* = 12)	GBM (IV), astrocytoma (III), oligodendroglioma (III)	3	MTR_asym_	APTw	Viable tumor core, edema, necrosis, mobile protein, and peptide	Wen Z. et al., 2010 [45]
Human (*n* = 14)	GBM (IV), AA (III), LGO (II), LGA (II), LGOA (II)	3	MTR_asym_	APTw	Protein content	Zhou J. et al., 2013 [42]
Human (*n* = 36)	GBM (IV), AO (III), AA (III), AOA (III), LGA (II), LGO (II), LGOA (II)	3	MTR_asym_	APTw	Glioma grading, necrosis, cell density, and proliferation	Togao O. et al., 2014 [39]
Human (*n* = 25)	Glioma (II–IV)	3	MTR_asym_	3 ppm	An acidic signature, treatment effect (CRT), and PFS	Harris RJ. et al., 2015 [38]
Human (*n* = 26)	GBM (IV), AA (III), AO (III), LGO (II), LGOA (II)	3	MTR_asym_	APTw	Glioma grading	Sakata A. et al., 2015 [65]
Human (*n* = 13)	GBM (IV), Gliomas (low–grade), meningiomas, lymphoma	3	MTR_asym_	APTw	NA	Togao O. et al., 2015 [36]
Human (*n* = 11)	High–grade glioma	3	EMR	APT, NOE	NA	Heo HY. et al., 2016 [119]
Human (*n* = 32)	High–grade glioma Lymphomas	3	MTR_asym_	APTw	Differentiate lymphomas from high-grade glioma and protein	Jiang S. et al., 2016 [64]
Human (*n* = 65)	Glioma (II–IV)	3	MTR_asym_	APTw	Proliferation	Park J. et al., 2016 [32]
Human (*n* = 32)	GBM (IV), AA (III), gliomas (low–grade)	3	MTR_asym_	APTw	Cellularity	Ma B. et al., 2016 [68]
Human (*n* = 65)	Glioma (II–IV)	3	MTR_asym_	APTw	Proliferation	Park J. et al., 2016 [32]
Human (*n* = 32)	GBM (IV), AA (III), gliomas (low–grade)	3	MTR_asym_	APTw	Cellularity	Ma B. et al., 2016 [68]
Human (*n* = 7)	AA (III), LGO (II), LGA (II)	3	MTR_asym_	APTw	NA	Zhang Y. et al., 2016 [88]
Human (*n* = 44)	Glioma (II–IV)	3	MTR_asym_	APTw	Glioma grading and proliferation	Bai Y. et al., 2017 [63]
Human (*n* = 46)	Glioma (II–IV)	3	MTR_asym_	APTw	Glioma grading, protein, and peptide	Choi YS. et al., 2017 [31]
Human (*n* = 24)	Glioma (II–IV), edema	3	MTR_asym_	APTw	Cellularity, proliferation, and glioma grading	Jiang S. et al., 2017 [30]
Human (*n* = 27)	Glioma (II)	3	MTR_asym_	APTw	IDH mutation	Jiang S. et al., 2017 [29]
Human (*n* = 42)	Glioma (II–IV)	3	MTR_asym_	APTw	Glioma grading, proliferation, choline, and *N*-acetylaspartate	Su C. et al., 2017 [27]
Human (*n* = 18)	GBM (IV)	3	MTR_asym_	APTw	MGMT promoter methylation status	Jiang S. et al., 2018 [24]
Human (*n* = 57)	Meningioma	3	MTR_asym_	APTw	Intracellular proteins and peptides	Joo B. et al., 2018 [23]
Human (*n* = 42)	Glioma (II–IV)	3	MTR_asym_	APTw	MGMT prediction	Su L. et al., 2018 [20]
Human (*n* = 21)	GBM (IV), glioma (II), metastases, meningoma, chronic infarction	3	MTR_asym_	APTw	Proteins and peptides	Sun H. et al., 2018 [120]
Human (*n* = 32)	Glioma (II–IV)	3	Z-spectral fitted,	APT	Glioma grading and proliferation	Zhang J. et al., 2018 [19]
			MTR_asym_	APTw		
Human (*n* = 51)	Glioma (II–IV)	3	MTR_asym_	APTw	Glioma grading and mobile cellular proteins	Zou T. et al., 2018 [62]
Human (*n* = 21)	GBM (IV), gliosarcoma (IV), AA (III),	3	MTR_asym_	APTw	Cellularity, proliferation, tumor recurrence, and a marker for active glioma	Jiang S. et al., 2019 [18]
Human (*n* = 71)	Glioma (III and IV)	3	MTR_asym_	APTw	Overall survival, PFS, and IDH mutation	Joo B. et al., 2019 [17]
Human (*n* = 14)	GBM (IV)	3	MTR_asym_	APTw	IDH and pH	Schure JR. et al., 2019 [108]
			Lorentzian	APT		
Human (*n* = 90)	Glioma (II–IV)	3	MTR_asym_	3 ppm	Cerebral blood volume and IDH mutation	Wang YL. et al., 2019 [72]
Human (*n* = 26)	Glioma (II, IV) Metastasis	3	MTR_asym_	APTw (3.5±0.4 ppm)	Glioma grading, MGMT, and IDH	Durmo F. et al., 2020 [61]
Human (*n* = 59)	Glioma (II, III)	3	MTR_asym_, machine learning	APTw	IDH1 mutation	Han Y. et al., 2020 [71]
Human (*n* = 54)	GBM (IV)	3	MTR_asym_	APTw	Treatment effect (bevacizumab), 12-month progression, PFS, and CBV	Park J. et al., 2020 [13]
Human (*n* = 30)	Glioma (III, IV)	3	MTR_asym_	APTw	Treatment effect (radiotherapy or CRT), tumor recurrence, and protein	Liu J. et al., 2020 [14]
Human (*n* = 46)	Glioma (II–IV)	3	MTR_asym_	APTw	Cellularity and CBV glioma grading	Schon S. et al., 2020 [59]
Human (*n* = 18)	GBM (IV), AA (III), astrocytoma (III), LGO (II), LGA (II)	3	MTR_asym_	APTw	Cytosolic protein content, mobile proteins, and semisolid macromolecules	Warnert EAH. et al., 2021 [11]
			Lorentzian	APT		
Human (*n* = 51)	Glioma (II–IV)	3	MTR_asym_	APTw	Glioma grading (peptide or protein concentrations), cellularity, proliferation, and IDH mutation	Xu Z. et al., 2021 [9]
Human (*n* = 48)	Glioma (II–IV), Brain metastases	3	MTR_asym_, machine learning	APTw	Protein content	Sartoretti E. et al., 2021 [12]
Human (*n* = 19)	GBM, meningioma, brain metastasis	3	QUASS	APT, MT&NOE (−1.5 ppm)	−1.5 ppm: proliferation	Wu Y. et al., 2021 [10]
Human (*n* = 48)	High–grade glioma (III,IV) Low–grade glioma (I,II)	3	CESTR^nr^, EMR	APT	Glioma grading (proteins and peptides)	Zhang H. et al., 2021 [8]
Human (*n* = 81)	H3K27M–mutant associated brainstem glioma	3	MTR_asym_	APTw	H3K27M mutation, proliferation, pH, and protein and peptide metabolism	Zhuo Z. et al., 2021 [6]
Human (*n* = 113)	Glioma (II–IV)	3	Lorentzian	APT	Glioma grading (cellularity, mobile protein, and peptides), and IDH mutation	Su C. et al., 2022 [5]
				2 ppm	Creatine and 1p/19q co-deletion	
Human (*n* = 1)	AA (III)	7	MTR_asym_	−3.5 ppm	Cellular density	Jones CK. et al., 2013 [44]
			Lorentzian	APT (3.3 to 3.7 ppm) NOE (−2 to −5 ppm)		
Human (*n* = 2)	GBM (IV), glioma (II or III)	7	MTR_asym_	−3 ppm	Necrosis and the structural integrity of proteins in cells (protein folding)	Zaiss M. et al., 2013 [121]
Human (*n* = 12)	GBM (IV)	7	MTR_asym_	3.3 ppm	Protein structures proliferation	Paech D. et al., 2014 [41]
Human (*n* = 15)	GBM (IV)	7	MTR_asym_	3.3 ppm	Cell density and edema	Paech D. et al., 2015 [37]
Human (*n* = 1)	LGO (II)	7	AREX	APT, NOE	NA	Windschuh J. et al., 2015 [35]
Human (*n* = 10)	GBM (IV)	7	AREX	3.5 ppm, NOE	Protein and lipid	Zaiss M. et al., 2015 [34]
Human (*n* = 10)	Gliomas (II–IV)	7	MTR_asym_	APTw	Glioma grading	Heo HY. et al., 2016 [33]
			EMR	APT (3.3 to 3.7 ppm) NOE (−3.3 to −3.7 ppm)		
Human (*n* = 11)	GBM (IV)	7	MTR_asym_, dnsAREX	3.5 ppm	Amide proton and pH	Zaiss M. et al., 2017 [25]
Human (*n* = 31)	Glioma (II–IV)	7	MTR_asym_, dnsAREX	APT (3.5 ppm)	Glioma grading, IDH mutation, and MGMT promoter methylation status	Paech D. et al., 2018 [22]
Human (*n* = 20)	GBM (IV)	7	Lorentzian	NOE	Treatment effect (First-line therapy)	Regnery S. et al., 2018 [21]
			MTR_asym_	APTw		
			dnsAREX	APT		
Human (*n* = 12)	GBM (IV), LGO (II), LGA (II)	7	AREX	NOE	Treatment effect (CRT)	Meissner JE. et al., 2019 [67]
			dnsAREX	APT		
Human (*n* = 26)	GBM (IV), AA (III)	7	AREX, dnsAREX	APT	Overall survival and PFS, amino acid, and protein	Paech D. et al., 2019 [16]
Human (*n* = 1)	GBM	9.4	Lorentzian	3.5 ppm, NOE (−1.6, −3.5 ppm), 2 ppm, 2.7 ppm	Proteins and lipids	Zaiss M. et al., 2018 [98]

B_0_, static magnetic field; GBM, glioblastoma; AO, anaplastic oligodendroglioma; AA, anaplastic astrocytoma; AOA, anaplastic oligoastrocytoma; LGO, low-grade oligodendroglioma; LGA, low-grade astrocytoma; LGOA, low-grade oligoastrocytoma; TMZ, temozolomide; CRT, chemoradiotherapy; PFS, progression-free survival; Cho/NAA, choline-to-*n*-acetyl-aspartate; I-IV: WHO classification tumor grade I-IV; CBV, cerebral blood volume; DISC, direct saturation-corrected; QUASS, quasi–steady-state. APTw refers to MTR_asym_ at 3.5 ppm, APT refers to CEST at 3.5 ppm, NOE refers to CEST at −3.5 ppm, unless the offset is specifically indicated.

### 2.3. Multiple CEST Contrast in Brain Tumors

Multiple CEST contrast could be beneficial for assessing the heterogenous molecular alterations in brain tumors. Other than the most studied CEST signals at 3.5 ppm and −3.5 ppm, other frequency offsets could provide valuable information within tumors, e.g., CEST contrast at 2 ppm was found to correlate with tumor aggressiveness [115,116]. An increased amine contrast at 2–3 ppm was observed in both animal [43,117] and human studies [38]. This could indicate the acidic microenvironment and an increase of amino acids in tumors which had low perfusion and a high level of hypoxia validated by cerebral blood flow (CBF) [38]. An increase of CEST contrast at 3 ppm could correspond to the increases in glutamate, glycine, and phenylalanine in tumors compared with contralateral regions [118]. These CEST contrasts could correlate with pH changes in tumors [38]. Amine/amide concentration independent detection (AACID) calculated based on an amide signal at 3.5 ppm and an amine signal at 2.75 ppm indicated the intracellular pH changes in mice at 9.4 T [66]. A recently published study including 113 brain tumor patients revealed changes in these multiple contrasts, including direct water saturation (DS), MTC, APT, NOE, and CEST at 2 ppm, reflected different tumor status and provided improved sensitivity and specificity in tumor diagnosis [5].

Moreover, multiple CEST contrast is reported to be sensitive to the treatment response. CEST at 3.5 ppm after anti-angiogenic therapy, radiation therapy, or chemoradiotherapy was decreased [13,14,21,32,40,66,67,68,69,70], but remained high in the case of tumor recurrence in rodents [70,92]. In patients, using more than one CEST contrast (e.g., APT and NOE) could identify the responders [21,67]. CEST at 3.5 ppm signal was reported to be well correlated with overall survival and progression-free survival [13,16,17], while CEST at −3.5 ppm signal was well correlated with overall survival [16]. In general, a change in CEST at 3.5 ppm and −3.5 ppm signal could be predictive for treatment responses and survival.

In summary, APTw contrast is unique in grading brain tumors at 3 T [27,30,39,60,64,65], although it could have multiple origins. It can be applied to study alterations in proteins, peptides, cellularity, proliferation, necrosis, IDH and MGMT, and metabolites. In addition to APT and NOE, other CEST contrasts have been explored to further study the underlying molecular alterations, including the regional alterations, in brain tumors, especially with the aid of radiomics [6,12].

## 3. Non-Metallic CEST Contrast Agents for Brain Tumor Imaging

While endogenous CEST contrast demonstrated uniqueness in imaging brain tumors, other non-metallic CEST contrast agents have been exploited to further enhance the contrast of specific molecular alterations in tumors [50,78,122,123,124,125]. Compared to endogenous molecules, the administration of contrast agents could provide a relatively high local concentration. Some contrast agents generate contrast further away from endogenous contrast, which typically exchanges at 1–4 ppm, could enhance the sensitivity by minimizing overlaps with background signals. The current clinical approach for brain tumor detection using MRI mainly relies on the use of gadolinium-based contrast agents (GBCAs) which do not enhance all types of tumors; in particular, the non-enhanced tumors could be malignant [126]. Moreover, it has safety concerns related to the development of incurable nephrogenic systematic fibrosis in renal compromised patients [127]. The Food and Drug Administration (FDA) also announced gadolinium retention in the brain could last for years [128,129].

**Figure 2:**
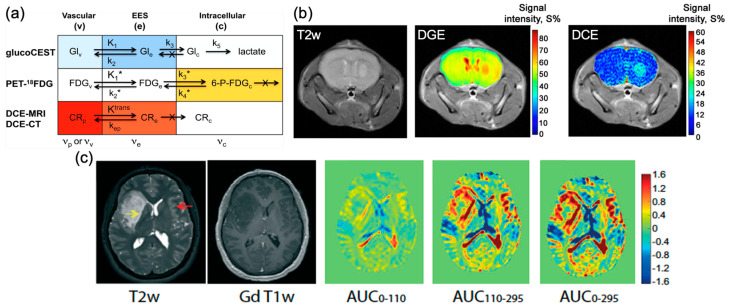
GlucoCEST and DGE MRI in brain tumors. (**a**) An overview of rate constants and contrast contributions (darker color = higher contrast; white is negligible contrast) for glucoCEST, ^18^FDG-PET, and contrast-enhanced MRI and CT in tumors. (**b**) T2-weighted image, DGE image at 300 s, and DCE image at 300 s for a mouse brain with tumor. (**c**) T2-weighted, gadolinium-T1-weighted, and DGE-based AUC images for different time periods (0–110 s, 110–295 s, 0–295 s) relative to the start of infusion for a human brain with glioma. Reproduced with permission from [130], John Wiley and Sons, 2012 [139], John Wiley and Sons, 2015 [140], MDPI, 2015.

One of the widely studied non-metallic CEST contrast agents for brain tumor imaging is D-glucose [123]. Glucose (C_6_H_12_O_6_) is a hexose with five hydroxyl (-OH) protons which can be detected by CEST MRI (Figure 2). In 2012, glucose was studied as a safe biocompatible nonmetallic contrast agent of CEST MRI [130,131,132,133,134] (Figure 2). Its hydroxyl protons generated CEST contrast at 0.8–2.2 ppm at 11.7 T (glucoCEST) [130,135]. In the first studies, researchers demonstrated that glucoCEST can be used to identify breast tumors and colon tumors in mouse models [130,135]. In breast tumors, glucoCEST is the only imaging modality in this study that could identify aggressive MDA-MB-231 tumors from less aggressive MCF-7 tumors, while conventional DCE-MRI using GBCAs and fluorodeoxyglucose (FDG) positron emission tomography (PET) did not show much difference. In the colon tumor study, researchers observed complementary signal changes in glucoCEST compared to FDG-PET. Regarding the uniqueness of breast tumor identification, the three-compartment model is proposed to explain the contributions to the signal changes (Figure 2a). D-glucose will participate in the three compartments, i.e., vasculature (v), EES, and intracellular space, while FDG is mainly distributed intracellularly. Once D-glucose goes intracellular, it is metabolized to lactate swiftly, as such, the detection of D-glucose in the EES of a tumor with low pH (~6.8 pH) could be more robust [130]. This was further illustrated in a brain glioblastoma mouse model (U87EGFRvIII) with inhibited hexokinase activity in which more glucoCEST contrast was detected in tumors with inhibition [136]. This could further support the major contributions from (v) and EES in tumors. Dynamic imaging of glucose was used in this study, i.e., dynamic glucose-enhanced (DGE) MRI [137,138,139,140]. DGE revealed a blood-brain-barrier (BBB) breakdown and an increased blood volume in brain tumors [139] (Figure 2b). It was translated to image brain tumor patients in 2015 [140] (Figure 2c), and can be applied to detect the BBB breakdown in patients at 3 T [141,142].

Other sugar analogs, such as 2-deoxy-D-glucose (2-DG) [143,144] and 3-O-Methyl-D-Glucose (3-OMG) [133,145], have been studied to image brain tumors in rodents. Notably, both 2-DG and 3-OMG cannot be metabolized, thus they mainly stay intracellularly. This could be similar to the distribution of FDG in PET imaging, which the tracer trapped intracellularly. Hence, this provides a wide imaging window for the intracellular compartment. Poly-L-glutamate could also enhance brain tumor imaging via generating CEST contrast at 3 ppm upon enzymatic cleavage in rat brain tumors [146]. Thus, many compounds could be repurposed as CEST contrast agents for brain tumor imaging.

## 4. Imaging Drugs and Drug Delivery

### 4.1. Imaging Drugs and Drug Delivery Using CEST MRI

**Figure 3:**
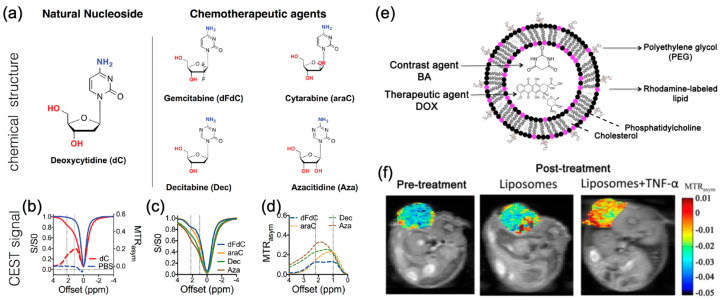
Contrast agent/drug-loaded liposome for CEST imaging. (**a**) The chemical structure of cytidine-based agents and (**b**–**d**) their CEST MRI contrast, as shown both by z-spectra (**b**,**c**) and MTR_asym_ plots (**b**,**d**). (**e**) Cartoon depicting the contrast agent/therapeutic agent (BA/DOX) co-loaded liposome. (**f**) MTR_asym_ maps at 5 ppm for a mouse bearing CT26 subcutaneous tumors before and after treatment with BA/DOX co-loaded liposome. Reproduced with permission from [147], Impact Journals, 2016 [153], Elsevier, 2014.

Many chemotherapeutics can be detected by CEST MRI. Liu et al. have shown that anticancer drugs, such as gemcitabine and their analogs, generated CEST contrast at 2–3 ppm in the presence of -OH and -NH_2_ exchangeable protons [147] (Figure 3a–d). Deoxycytidine was phosphorylated by deoxycytidine kinase in cells, thus imaging deoxycytidine could indicate this enzymatic activity [84]. Melphalan with -NH_2_ exchangeable protons is another potential drug candidate for brain tumor treatment that can be detected by CEST at 2.5 ppm [147].

Imaging drug delivery has implications in the design of drug carriers and detection of the amount of drugs delivered to tumors, thereby assessing the therapeutic effects. Liposomes are versatile carriers for both contrast agents and drugs. They were approved as a carrier for doxorubicin, a chemotherapeutic in 1995 [148], and the generic version was approved in 2013. Doxil reaches tumors based on the enhanced permeability and retention (EPR) effect to passively target tumors. Liposomes have been used as carriers for non-metallic CEST contrast agents [51,122,124,149,150,151,152]. Liposomes containing barbituric acid (BA) CEST contrast agents could target tumors based on EPR [153,154,155] (Figure 3e), and reveal the therapeutic effect of tumor necrosis factor-alpha (TNF-α) (Figure 3f), which induce hyperpermeability in vasculature and destruction of the vascular lining [153]. However, the presence of the BBB could limit the delivery of drug-containing liposomes to brain tumors. In the considerations of the presence of the BBB and the efficacy of drugs under physiological environment, we recently developed a CEST MRI-guided nose-to-brain drug delivery system based on CEST detectable liposomes [156]. The benefit of intranasal drug delivery is that it can bypass the BBB and hence enables relatively high dose of drugs reaching the brain. There are two clinical trials underway for intranasal delivery of chemotherapeutics to the GBM (NCT04091503; NCT02704858).

### 4.2. Theranostic Applications

**Figure 4:**
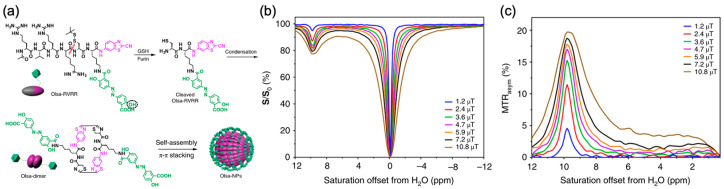
(**a**) Schematic illustration for the formation of Olsa-NPs by furin-mediated intracellular reduction and condensation of Olsa-RVRR; (**b**) Z-spectra and (**c**) MTR_asym_ values of 10 mM olsalazine for different saturation powers. Olsa: olsalazine; NPs: nanoparticles. Reproduced with permission from [157], Springer Nature, 2019.

To further extend the benefit of CEST in detecting molecules/drugs with their natural labels to theranostic applications, Yuan et al. designed cell-penetrating and self-assembling olsalazine nanoparticles. Olsalazine is a chemotherapeutic agent having hydroxyl protons that exchange at 9.8 ppm. The molecular structure and sequence of reactions are shown in Figure 4a [157]. In brief, in the presence of glutathionine (GSH) and furin, the olsalazine (Olsa)-RVRR will form self-assembled particles in cells. In mice bearing both high and low furin expressing tumors, the tumor with high furin expression had a significantly higher CEST contrast at 9.8 ppm compared to the tumor with low furin expression (Figure 4b) [157]. CEST contrast at 9.8 ppm is unique for Olsalazine and did not overlap with endogenous CEST contrast. The increase in this CEST contrast over time indicates the accumulation of these drug nanoparticles in tumors.

**Figure 5:**
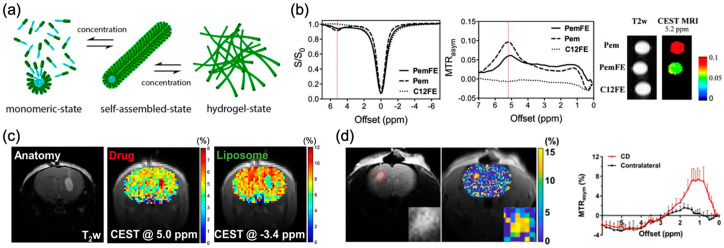
(**a**) Schematic illustration of the self-assembly of PemFE monomers into filamentous nanostructures that can further entangle into a 3D network for the formation of self-supporting hydrogels under suitable conditions (pH, concentration, and ionic strength). (**b**) Z-spectra, MTR_asym_ plots, and CEST contrast maps of PemFE (solid), Pem (dashed), and C12FE (dotted) showing CEST peaks at 5.2 ppm. (**c**) T2-weighted image and CEST images (5.0 ppm and −3.4 ppm) of drug barbituric acid (BA) loaded liposomal hydrogels in a mouse brain. (**d**) T2-weighted image, MTR_aysm_ image at 1.1 ppm, and the average MTR_aysm_ spectra of CD hydrogel in a mouse brain. Pem: Pemetrexed. Reproduced with permission from [159], American Chemical Society, 2017 [166], Ivysrping International Publisher, 2020 [167], American Chemical Society, 2021.

Other than nanoparticles, a hydrogel is another potential biomaterial for drug delivery to brain tumors. The CEST imaging of a hydrogel incorporated with liposomes as a pH-nanosensor indicated the potential to detect small and local changes in pH for detecting cell viability in vivo [158]. Pemetrexed (Pem), which is an anticancer drug with -NH_2_ (heterocyclic ring amide), conjugated with glutamic acid and phenylalanine and formed a nanofiber hydrogel that generates CEST contrast at 5.2 ppm in a mouse brain via local injection (Figure 5) [159]. The current adjuvant treatment after brain tumor resection is to place a carmustine wafer at the tumor resection site [160,161]. Its wide clinical application has been hindered by the side effect of wafer degradation. Studies have demonstrated the potential of using a hydrogel instead of the wafer for local drug delivery to brain tumor resection sites [162,163,164]; such a hydrogel loaded with paclitaxel showed a controlled and sustainable release of the drug over 6 weeks after injection in the proximity of the GBM in mice [162,165]. In another hydrogel study, Han et al. showed that an injectable liposomal hydrogel generated CEST contrast at both 5 ppm and −3.4 ppm, which indicate the intraliposomal drug barbituric acid (BA) and liposomes, respectively. This is the first use of CEST contrast at −3.4 ppm for liposome imaging at 3 T [166]. Interestingly, the release at 5 ppm is relatively faster than that at −3.4 ppm, indicating a different release profile between intraliposomal drugs and liposomes. This demonstrates the need to image both drug and carrier simultaneously and independently (Figure 5c). Another study in 2021 showed CEST imaging of a self-healing hydrogel loaded with gemcitabine (Gem) at 2.2 ppm which exhibited sustainable cytotoxicity towards the human glioblastomas cell line [167]. This chitosan-dextran (CD) based hydrogel generated inherent CEST contrast at 1.1 ppm at 3 T MRI (Figure 5d). These CEST detectable chemotherapeutics and hydrogels could be promising approaches for controlled drug release and local treatment of brain tumors under MRI guidance.

## 5. Technical Part

CEST contrast is dependent on acquisitions and post-processing methods. In brain tumors, careful interpretation is needed since other contributions, such as MT and T1, could be quite different from normal brain tissues [10,21,25,34,89,90,96,99,100,104,168,169]. In this section, we will explain the principle of CEST acquisition, the common methods in analyzing the Z-spectrum, and recent developments in using deep-learning to assist CEST post-processing.

### 5.1. CEST Acquisition

The CEST signal is acquired by detecting the water signal after saturation of exchangeable protons of the molecules (solute) using frequency selective radio frequency (RF) pulses. This signal reduction of the exchangeable protons will then be transferred to the bulk water protons at 110 M [49,50,51]. The exchange process can be quantified by the exchange rate of ksw from solute to water (or kws from the water back to solute). After saturation, the water signal is measured by common MRI sequences. The difference between saturated and unsaturated water signals can be used to quantify the solute. In typical Z-spectrum acquisition, the frequency of saturation RF pulse is swept at a range with offsets at both sides of the water signal (defined at 0 ppm), depending on the types of exchangeable protons [170]. This Z-spectrum is slightly different from conventional MRS or NMR where the water peak is at 4.7 ppm with respect to tetramethylsilane (TMS). The saturated water signal (S_sat_) is usually normalized to the unsaturated water signal (S_0_). By analyzing the Z-spectrum, the CEST contrast of the molecule can be obtained.

The basic CEST sequence consists of a pre-saturation module and an image readout module [46,50,52,169]. The pre-saturation module is to label the exchanging protons using an irradiation field with an amplitude of B_1_, a duration of tsat, and a frequency offset of Δω with respect to water signal at 0 ppm. The B_1_ and tsat are closely related to CEST sensitivity and typically require optimization for a specific CEST study. In the CEST field, the commonly used saturation module is either a continuous-wave CEST/spin lock or pulsed CEST [52], while a pulsed CEST is currently more preferred in clinical applications because of special absorption rate (SAR) limitations. Pulsed CEST has another advantage of suppressing the MT effect [81,97]. Theoretically, the readout module can be any MRI sequence that includes excitation, spatial encoding, and data acquisition. With considerations of both signal-to-noise ratio (SNR) and scan time, the commonly used image readout module in CEST is turbo spin echo (TSE), also known as fast spin echo (FSE) or rapid acquisition with relaxation enhancement (RARE).

### 5.2. CEST Post-Processing

#### 5.2.1. Z-Spectra and B_0_/B_1_ Correction

CEST MRI requires specific post-processing to obtain contrasts. After acquiring CEST data, Z-spectra can be obtained by following equation [50,52]:

(1) $$Z\left(\Delta \omega \right)=\frac{S_{sat}\left(\Delta \omega \right)}{S_{0}},$$

where Δω represents the frequency offset with respect to the water frequency at 0 ppm, $S_{sat}\left(\Delta \omega \right)$ and $S_{0}$ are the steady-state magnetization with saturation at Δω and without saturation, respectively. Since the quantification of Z-spectra is exquisitely sensitive to static magnetic field (B_0_) inhomogeneity, which exists in most MRI scanners, B_0_ correction is needed. Typically B_0_ correction is performed in two steps: (i) Generate a B_0_ map: the B_0_ map can be obtained by estimating the minimum of interpolated/fitted Z-spectra [48,171,172], water saturation shift referencing (WASSR) [173], or other B_0_ mapping methods [174,175,176]. The latter two require extra data acquisition in addition to CEST data. (ii) Correct B_0_ inhomogeneity for Z-spectra: the frequency shift values on B_0_ map are applied to correct the corresponding Z-spectra on a pixel-by-pixel basis. After B_0_ correction, it is sometimes suggested performing B_1_ correction if the scanner has large B_1_ inhomogeneity [35,176]. Similar to B_0_ correction, B_1_ correction includes two steps: (i) Generate B_1_ map: the B_1_ map can be acquired using flip-angle mapping [35,177], double angle method (DAM) [174,178], or other B_1_ mapping methods [175,176]. (ii) Correct B_0_ inhomogeneity for Z-spectra or CEST contrasts: the relative values on B_1_ map are applied to correct the corresponding Z-spectra or CEST contrasts on a pixel-by-pixel basis. Notably, whether to correct B_0_ or B_1_ depends on the scanner used for data collection and the analysis method used to extracted CEST contrasts. Finally, the Z-spectra are analyzed to obtain the CEST contrast. Z-spectra analysis methods can be categorized into three types, i.e., Z-spectra analysis, Inverse Z-spectra analysis, and deep-learning methods.

#### 5.2.2. Z-Spectra Analysis

Conventional Z-spectrum analysis can be summarized as quantifying the CEST contrasts using the difference between the acquired Z-spectrum (Z) from the reference spectrum (*Z_ref_*). Currently, many methods, including MTR_asym_ [47,48], Lorentzian difference (LD) [44,179], multi-pool Lorentzian fitting [180,181], polynomial and Lorentzian line-shape fitting (PLOF) [182,183,184], and three-offset method [93,185,186] have been applied in different CEST studies.

(1) *MTR_asym_* analysis

*MTR_asym_* analysis directly uses the signal at symmetrical frequency offsets in water frequency on *Z*-spectra as a reference to calculate the CEST contrasts [47,48]:

(2) $$MTR_{asym}\left(\Delta \omega \right)=Z_{ref}\left(\Delta \omega \right)-Z\left(\Delta \omega \right)=Z\left(-\Delta \omega \right)-Z\left(\Delta \omega \right).$$

This method is more suitable for analyzing CEST data without CEST/MT on the negative side of Z-spectra, such as solution phantom data. When it is applied in analyzing in vivo data, the calculated result should be interpreted carefully as it contains multiple molecular contributions from both sides of the Z-spectrum, such as asymmetric MTC.

(2) Lorentzian difference analysis (LDA)

LDA first fits the *DS* effect using a Lorentzian line shape to obtain the reference spectra [44,179]:

(3) $$Z_{ref}\left(\Delta \omega \right)=1-L_{DS}=1-\frac{A_{DS}}{1+{\left[\frac{\Delta \omega -\delta_{DS}}{\Gamma_{DS}/2}\right]}^{2}},$$

and then subtracts the acquired Z-spectrum from the fitted reference curve to obtain the CEST contrasts:

(4) $$CEST\left(\Delta \omega \right)=Z_{ref}\left(\Delta \omega \right)-Z\left(\Delta \omega \right)=1-\frac{A_{DS}}{1+{\left[\frac{\Delta \omega -\delta_{DS}}{\Gamma_{DS}/2}\right]}^{2}}-Z\left(\Delta \omega \right),$$

where A*_DS_* represents the water peak amplitude, Γ*_DS_* is the full-width-at-half-maximum (FWHM) of water peak, and δ*_DS_* is the water peak position (can be set to 0 ppm after B_0_ correction). For in vivo study, MT contrast may affect interested CEST contrasts (especially NOE) and thus can be excluded from the *Z_ref_*. In this case, an MT term (*L_MT_*) is additionally subtracted from Equation (3) [187].

(3) Multi-pool Lorentzian fitting

Multi-pool Lorentzian fitting is another Lorentzian-based method for analyzing the Z-spectrum [180]. With each pool taken as a Lorentzian line shape, the fitted Z-spectrum can be expressed as:

(5) $$Z_{fit}\left(\Delta \omega \right)=1-\sum_{i=1}^{n}L_{i}\left(\Delta \omega \right).$$

*L_i_* refers to all peaks that contribute to the Z-spectra, including all CEST, DS, and MT peaks:

(6) $$L_{i}\left(\Delta \omega \right)\;=\;\frac{A_{i}}{1+{\left(\frac{\Delta \omega -\delta_{i}}{\Gamma_{i}/2}\right)}^{2}}$$

where A*_i_*, Γ*_i,_* and δ*_i_* refer to the amplitude, FWHM, and position of each peak, respectively. For multi-pool Lorentzian fitting, the *Z_ref_* of pool *i* can be expressed as:

(7) $$Z_{ref,i}\left(\Delta \omega \right)=1-\sum_{j\neq i}^{n}L_{j}\left(\Delta \omega \right),\;\left\{1\leq j\leq n\right\}.\;$$

It is worth noting that the initial values and boundary values may affect the accuracy of multi-pool Lorentzian fitting and thus need to be defined properly [181]. At low field strength, some CEST peaks overlap with adjacent peaks, thus the total pool numbers need to be adjusted accordingly [99,188].

(4) Polynomial and Lorentzian line-shape fitting (PLOF)

PLOF is a CEST analysis method that combines Lorentzian fitting for CEST signal (*R_ex_*) with N-order polynomial fitting for background signal (*R_back_*) [182,183,184]:

(8) $$R_{ex}\left(\Delta \omega \right)=\frac{R_{exch}^{max}}{1+{\left(\frac{\Delta \omega -\delta }{\Gamma_{i}/2}\right)}^{2}},\;$$

(9) $$R_{back}\left(\Delta \omega \right)=\overset{N}{\underset{n=1}{\sum }}C_{n}{\left(\Delta \omega -\delta \right)}^{n},\;$$

where *C_n_* is the coefficient of the *n*-th order. After fitting, the observed CEST contrast is calculated by:

(10) $$CEST\left(\Delta \omega \right)=Z\left(R_{eff}+R_{back}\right)-Z\left(R_{eff}+R_{back}+R_{ex}\right)$$

where *R_eff_* is the measured longitudinal relaxation rate of water in the rotating frame without additional solutes. Here, the *Z*(*R_eff_* + *R_back_*) can be taken as reference signal Zref while the *Z*(*R_eff_* + *R_back_* + *R_ex_*) can be taken as labeled signal *Z_lab_*. Currently, PLOF is mainly used to analyze creatine CEST data; it is also applicable to amide CEST with adjustment on background signal fitting [175].

(5) Three-offset method

Some studies reported a simple three-offset method to obtain APT and NOE CEST contrasts [93,185,186], thus reducing the scan time by skipping data acquisition of some frequency offsets. The CEST contrast is calculated by:

(11) $$CEST\left(\Delta \omega \right)=Z_{ref}\left(\Delta \omega \right)-Z\left(\Delta \omega \right)=\frac{Z\left(\Delta \omega -\delta \omega \right)+Z\left(\Delta \omega +\delta \omega \right)}{2}-Z\left(\Delta \omega \right)$$

where δω refers to the frequency offset with respect to Δω and can be set to different values for different CEST contrast at different field strengths. The three-offset method is more suitable for CEST studies at high fields as it relies on a clear delineation of CEST peaks [185].

#### 5.2.3. Inverse Z-Spectra Analysis

Endogenous CEST effects close to 0 ppm are easily attenuated by the DS and MT effects. Zaiss et al. proposed to analyze Z-spectrum inversely to obtain the CEST contrasts with correction of these effects [34,189]:

(12) $$CEST_{Rex}=\frac{1}{Z_{lab}}-\frac{1}{Z_{ref,\;i}},$$

where *Z_lab_* and *Z_ref_* represent the Z-spectrum values of label and reference, respectively. The *Z_ref_* can be calculated using the above-mentioned five methods in Section 5.2.2, the *Z_lab_* is the acquired (labeled) Z-spectra data. Moreover, CEST contrast is also affected by the longitudinal relaxation time (T1) of water. To address this problem, AREX contrast, which excludes T1 contributions, can be used to calculate the CEST signal that more represents chemical exchange than conventional CEST analysis [34,189]. The AREX contrast is calculated by:

(13) $$AREX=CEST_{Rex}\cdot R_{1}=\left(\frac{1}{Z_{lab}}-\frac{1}{Z_{ref,\;i}}\right)\cdot R_{1},$$

where R1 (1/T1) refers to the longitudinal relaxation rate.

#### 5.2.4. Deep Learning-Based Analysis Methods

Recently, deep learning-based methods [15,99,100,104,188,190,191] have also been applied to obtain CEST contrasts to speed up and simplify the post-processing of CEST. Deep learning utilizes neural networks that are composed of multiple processing layers to extract information from data [192].

(1) Deep learning-based Z-spectra analysis

In the presence of the scaling effects from the MT effect and DS, some CEST peaks cannot be clearly identified, especially at a low magnetic field (3 T or less). Zaiss et al. proposed to predict 9.4 T CEST signals from 3 T CEST signals using a deep neural network (deepCEST), demonstrating the feasibility of extracting unobservable CEST signals from low-field CEST data [100]. Glang et al. further improved the performance of deepCEST at 3 T using a probabilistic deep learning approach which provides additional information about the reliability of the prediction [99]. In addition to deepCEST, Huang et al. demonstrated that the AREX contrasts can be predicted by a deep neural network (deepAREX) which requires both Z-spectra and T1 as inputs [188]. Chen et al. utilized an artificial neural network to extract CEST properties (ANNCEST), such as metabolite concentration, exchange rate, and B_0_/B_1_. The ANNCEST is sensitive to map the phosphocreatine (PCr) concentration in human skeletal muscle on a 3 T clinical scanner [15].

(2) Deep learning-based CEST fingerprinting

Some other studies combined MR fingerprinting with a deep neural network reconstruction to obtain the molecular properties, including concentration and exchange rate [104,190,191,193]. In this case, the CEST sequence needs to be specifically designed and a simulation dictionary should be built for network training.

Despite the differences in the technical details of these methods, all the deep learning-based methods could substantially speed up the post-processing of CEST MRI (e.g., up to a second level after training). These studies all demonstrated the great potential of deep learning-based CEST analysis.

## 6. Promises and Challenges

CEST imaging of amide protons at 3.5 ppm has been successfully translated to image brain tumor patients in a short period of time due to its non-invasive nature. Other CEST contrasts indicate alterations of aliphatic protons (NOE, typically at −3.5 ppm) and amine protons (at 2 ppm) could be considered collectively in diagnosis, prognosis, and assessment of the treatment effects in brain tumors. Moreover, CEST is also capable of detecting drugs approved for clinical use, such as anticancer drugs. This detection of natural labels on molecules could facilitate the development of image-guided drug delivery to the brain, especially those that can cross or bypass the BBB. Theranostic approaches could facilitate the assessment of treatment effects longitudinally and multiple component changes in drug delivery systems, such as liposomes and hydrogels.

In addition to CEST MRI, various imaging approaches provide valuable information to characterize the heterogeneity of brain tumors. For example, diffusion weighted imaging (DWI) or diffusion tensor imaging (DTI) measures the degree of water movement, thus detecting pathologies that change this movement in brain tumors [31,194,195]. Perfusion weighted imaging (PWI) using DCE, dynamic susceptibility contrast (DSC), and arterial spin labeling (ASL) is commonly used to measure cerebral hemodynamics [111,196,197,198,199]. Both diffusion and perfusion indicate structural abnormalities in tumors, such as leaky vasculature and poor drainage [200,201]. Proton ^1^H MRS has been used to evaluate the altered metabolism in brain tumors [202,203], though its application is limited by the limited spatial information. Other than MRI, FDG-PET is a commonly used imaging approach for tumor detection and grading as tumors typically have abnormal glucose uptake [204,205]. Compared to these clinical approaches, CEST is a sensitive and non-invasive method of detecting molecular alterations in terms of concentration and exchange environment. Majority MRI approaches characterize tumors with respect to structural alterations, FDG-PET is regarded as an invasive approach whose pros and cons can be found in Section 3. Moreover, DWI, DTI, ASL, MRS, and CEST do not require the administration of contrast agents which could support non-invasive and frequent assessments. Thus, DWI/DTI and ASL could be applied in conjunction with CEST to further characterize heterogenous brain tumors.

This uniqueness of CEST MRI has led to enthusiasm in the field, but challenges remain in clinical translations in both data acquisition and post-processing. First, B_1_ needs to be optimized to achieve a high labeling efficiency during acquisition for exchanging protons and the RF constraint, i.e., specific absorption rate (SAR), especially at low B_0_ field. Second, the CEST sequence also needs optimization because Z-spectrum acquisition requires measurements at a series of frequency offsets, which is time-consuming. Third, motion correction approaches are needed for human applications as small motion could induce error in CEST quantification. Fourth, there is no standardized method yet to analyze CEST data and there are many post-processing methods available. Finally, the interpretation of changes in CEST contrast is not straightforward. Great caution should be taken as CEST contrast depends on the molecular alterations in vivo, such as concentration and exchange rate. Moreover, brain tumors have complicated and heterogenous microenvironments, such as liquefactive necrosis and hemorrhage. Researchers in the field are working together to standardize the CEST imaging of brain tumors. Recent developments in pulse sequences [175,206,207,208,209,210] that strive to achieve high SNR, whole-brain coverage, and short acquisition time, together with the deep learning-based analysis, are expected to open new avenues for realizing fast and accurate CEST MRI.

## 7. Conclusions

CEST MRI is a promising non-invasive imaging method to detect molecules at the millimolar level. Great promise has been shown in the grading of brain tumors, potential identification mutation, and regional changes, such as radiation necrosis, cellularity, and IDH. CEST MRI also enables the detection of chemotherapeutics, liposome-based, and hydrogel-based drug delivery. These theranostic applications could provide valuable information for the adjustment of treatments, demonstrating the potential for precision medicine. Both the acquisition and post-processing of CEST are not so trivial and could be addressed by using a specific pulse sequence design and deep learning-based analysis. When more preclinical and clinical studies become available, technical hurdles may be overcome to bring this promising field to wide neuroimaging applications.

## Data Availability

Not applicable.
